# A Randomized Controlled Trial of Misoprostol and Sulprostone to End Pregnancy after Fetal Death

**DOI:** 10.1155/2009/496320

**Published:** 2009-09-06

**Authors:** Kristin Van Mensel, Filip Claerhout, Patrick Debois, Marc J. N. C. Keirse, Myriam Hanssens

**Affiliations:** ^1^Women and Children Division, Department of Obstetrics and Gynaecology, University Hospital Gasthuisberg, 3000 Leuven, Belgium; ^2^Department of Obstetrics, Gynaecology, and Reproductive Medicine, Flinders University, Bedford Park, Adelaide, SA 5042, Australia

## Abstract

*Objective*. To compare effectiveness, side effects, and patients' perception of vaginal misoprostol *versus* intravenous sulprostone for ending pregnancy after fetal death between 14 and 42 weeks gestation. *Method*. Multicenter randomized controlled trial, using block randomization, central allocation, and prior power analysis. *Outcome measures*. Induction-delivery interval, gastrointestinal side effects, use of analgesia, pain perception, pyrexia, placental retention, hemorrhage, and women's opinions. *Results*. Of 176 women aimed for, 143 were randomized over 7 years, of whom 4 were excluded. There was no difference in delivery within 24 and 36 hours: 91.4% and 97.1% with misoprostol (*n* = 70) *versus* 85.5% and 92.8% with sulprostone (*n* = 69). There was no difference in either gastrointestinal side effects, as reported by the women and their caregivers, use of analgesia, women's pain perception, blood loss or placental retention. Hyperthermia ≥38°C was more common with misoprostol (24.3%) than with sulprostone (11.6%; difference: +12.7%; 95% CI: +1.2% to +25.3%) and related to the total dose used. Acceptability of both induction methods was similar except for freedom of movement, which was substantially in favor of misoprostol (lack of freedom reported with misoprostol in 34.3% *versus* 63.8% with sulprostone; difference: −29.5%; 95% CI: −13.6% to −45.4%). *Conclusions*. Misoprostol and sulprostone are similarly effective with little difference in side effects except for hyperthermia, related to the dose of misoprostol used, and women's reported lack of mobility with intravenous sulprostone. Effectiveness of both methods increased with gestational age.

## 1. Introduction

Intrauterine fetal death is a rare event after the first trimester of pregnancy. Retention of the dead fetus may cause consumptive coagulopathy, but this is a rare complication occurring after a prolonged retention of several weeks [1, 2]. As there is rarely a medical need to end the pregnancy, the emotional distress of carrying a dead baby is usually the sole reason for intervention in such circumstances. It is important that the intervention not only achieves what it aims to achieve but also does so with as little distress and discomfort as possible. Traditional induction methods, such as amniotomy and oxytocin, are notoriously ineffective, particurlarly at low gestational ages as there is a 20-fold difference in oxytocin sensitivity between mid-pregnancy and term [3, 4]. As reviewed by Keirse [5], before the advent of prostaglandins [6], inducing labor after early fetal death was frequently an ordeal for both the patient and her caregivers. Of the many treatments used, high doses of oxytocin were the most effective [7], but these nearly always led to some degree of water retention [8], often requiring the treatment to be spread over several days to avoid its consequences [5]. Combining the use of oxytocin with amniotomy could reduce the induction-expulsion interval, but exposed the woman to infection, particularly when the treatment was ineffective [9].

Prostaglandins and prostaglandin analogues are known to be effective, even more effective after fetal death than they are in pregnancies with a live fetus at similar pregnancy duration [5, 10, 11]. However, in the doses required well before term, they are associated with unpleasant side effects especially of the gastrointestinal tract [12–14]. Sulprostone is a PGE_2_ analogue with a greater uteroselectivity than the parent compound [15] and, therefore, fewer gastrointestinal side effects. At the time this study was started, it was the most commonly used agent for induction of labour after fetal death in the Netherlands, with its dose regimen for that indication well established from a randomized controlled trial [14]. We compared this treatment with sulprostone, a PGE_2_ analogue registered for obstetric use, with the use of misoprostol, a PGE_1_ analogue. Although not registered for obstetric use and marketed for the prevention and treatment of gastroduodenal ulcers, misoprostol was becoming widely used for a variety of obstetric indications and was purported to cause, in the doses required, fewer systemic side effects than other prostaglandin preparations. A multicenter randomized controlled trial was initiated to evaluate the relative merits of misoprostol and sulprostone.

## 2. Patients and Methods

All women with a fetal death between 14 and 42 weeks of gestation at one of 12 participating hospitals were eligible for the study provided they were not in labor, had no placental abruption, no problems of communication, and gave informed consent. The study protocol was approved by the ethical committee.

The 1 to 1 randomization sequence was computer generated in blocks of 10 and 20. Randomization was centrally controlled and allocation was by phoning the central unit where a serially numbered, sealed, opaque envelope was opened for each participant. While concealment of allocation was ensured, once allocated no attempt was made to blind women or caregivers to the actual treatment, as this would have required additional placebo interventions in all women either intravenously or vaginally.

Sulprostone was administered intravenously, as previously evaluated in a randomized controlled trial [14], at 1 *μ*g/min (1 ampoule of 500 *μ*g sulprostone in 250 mL NaCl 0.9%, at 30 mL/min) for 36 hours or until fetal and placental expulsion, if this occurred earlier. As this low dose was administered by continuous intravenous infusion, no adjustment for gestational age was deemed necessary [14]. Misoprostol doses, on the other hand, were based on gestational age: 400 *μ*g between 14 and 26 weeks, 100 *μ*g between 27 and 36 weeks, and 50 *μ*g between 37 and 42 weeks. While adjusting doses of misoprostol according to gestational age corresponds with the known increase in uterine sensitivity to prostaglandins with advancing gestation [5] and has become common practice since [16], this regimen was based on anecdotal evidence at the time. Misoprostol was administered in the posterior vaginal fornix at 4-hourly intervals up to a maximum of 4 doses per 24 hours or until labor was established. Intravenous oxytocin was started if augmentation of labor was deemed necessary. In both treatment arms women were switched to the other regimen after 36 hours, if complete expulsion had not occurred within 36 hours after the start of treatment.

Gastrointestinal side effects, severe vomiting and diarrhea in particular, were the primary outcomes of interest. Sample size was determined on the basis of an anticipated 4-fold reduction in the frequency of these side effects from 20% in the sulprostone group [14] to 5% with misoprostol. This required 76 patients per group given an *α* error of 0.05 (two-sided) and a *β* error of 0.2. We anticipated to recruit 88 women per group to allow for a 15% drop-out or switching to the alternative treatment. Induction-delivery interval, use of analgesia, estimated blood loss, need for additional intervention, and measures of maternal satisfaction were the other predetermined outcome measures.

Side effects, pain, nausea, vomiting, and diarrhea, were scored independently by the midwife caring for the woman and by the women themselves. Midwives monitored side effects during the procedure and scored these on the basis of the perceived need for and use of additional medications. Thus, pain was scored as no pain relief needed, mild oral analgesics, intramuscular narcotics, and epidural analgesia. Nausea and vomiting were scored as no nausea, light no drugs required, need for antiemetics, and vomiting not remedied by antiemetics. Diarrhea was similarly scored as no diarrhea, light no drugs required, and severe enough to warrant medication.

Women scored side effects independently after having left the labor ward. Pain experience was scored on a visual analogue scale from zero to ten. Women also indicated what pain relief they had received and whether they had to wait too long for pain relief. Nausea and diarrhea were expressed on a scale from zero to three (none, light, average, or heavy). Vomiting was scored as yes or no and the number of episodes. Women's opinions were also sought on whether they had felt restricted in their mobility, how they assessed the duration of the procedure and whether they were generally satisfied or would have preferred another management.

Differences in outcome between groups were assessed with the chi-square test and confidence interval (CI) analysis.

## 3. Results

Only 143 of the a priori determined number of 176 women were randomized over a period of 7 years, before recruitment was stopped. Cessation of recruitment was prompted by the fact that even without formal data analysis, clinicians in most centers had become increasingly convinced of the superiority of misoprostol over sulprostone to the extent that they could no longer justify randomization between the two treatments.

**Figure 1:**
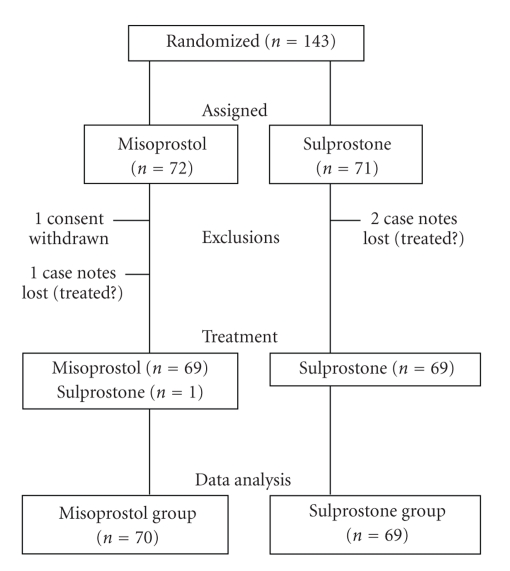
Flow diagram from randomization to analysis.

Four of the 143 women (2.8%) were excluded from the analysis (Figure 1). One, assigned to misoprostol, withdrew her consent after randomization. Case notes were lost for 3 others (1 in the misoprostol and 2 in the sulprostone group) and it could not be ascertained whether they actually had or had not received the allocated treatment or had given birth spontaneously. One woman in the misoprostol group was started on the alternative treatment to what she had been allocated. She was retained in her assigned group. Baseline characteristics of the 139 women remaining in the study are shown in Table 1.

**Figure 2:**
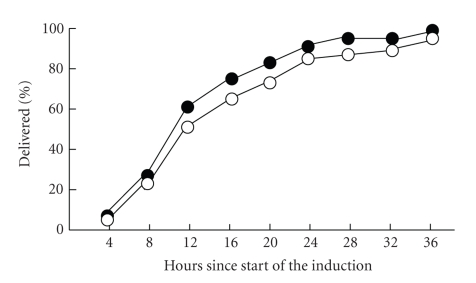
Cumulative delivery rate in women assigned to vaginal misoprostol (closed circles) or intravenous sulprostone (open circles).

The median induction-delivery interval was 2 hours shorter with misoprostol than with sulprostone (Table 2, Figure 2), but the difference was compatible with chance. Including one woman in the sulprostone group, who was switched to misoprostol before the scheduled 36 hours because of pronounced dilatation of the antecubital vasa vasorum, the need to resort to the alternative treatment before complete expulsion, was similar in both groups (4.3% with misoprostol *versus* 8.7% with sulprostone; difference: −4.4%; 95% CI: −12.6% to +3.8%). So was the use of additional oxytocic agents (difference: +4.0%; 95% CI: −10.5% to +18.4%; Table 2). The frequency of placental retention for 1 hour or more (difference: +4.1%; 95% CI: −8.7% to +16.8%) and the need for curettage or manual exploration (difference: +3.8%; 95% CI: −11.9% to +19.5%) were also similar in both groups (Table 2).

Placental retention, whilst not related to treatment allocation, was clearly associated with low gestational age. Of 25 women with placental retention for >1 hour (14 in the misoprostol and 11 in the sulprostone group; Table 2), 23 were under 22 weeks. Placental retention occurred in 32% of women (23 of 72) under 22 weeks compared with less than 5% (2 of 43) at 28 weeks or more (difference: 27%; 95% CI: 14.8% to 39.8%). Similarly, 42 of 47 women who required curettage or manual removal were under 28 weeks. Overall 60% (42 of 70) of women in the misoprostol group had the placenta delivered spontaneously within 1 hour without curettage or manual removal as compared to 63.8% in the sulprostone group (difference −3.8%; 95% CI: −19.9% to +12.4%; Table 2).

Whilst postpartum hemorrhage of 1000 mL or more occurred in 8% (11 of 139) with no difference between the two groups (Table 2), blood transfusion was given to one woman treated with misoprostol and three treated with sulprostone (difference: −2.9%; 95% CI: −8.4% to +2.6%).

Our prior hypothesis of a lower rate of gastrointestinal side effects among misoprostol treated women was not confirmed. Gastrointestinal side effects, whether reported by the caregivers (Table 3) or by the women (Table 4), occurred with a similar frequency and similar severity in both groups. There are indications for a difference in the perception of gastrointestinal side effects between women and their caregivers. Whereas 10% of the women in the misoprostol group found that they had severe nausea and 15.7% reported several episodes of vomiting, only 4.3% had received antiemetic treatment. Comparable figures in the sulprostone group were, respectively, 2.9%, 15.9%, and 8.7%. Severe diarrhea was reported by 1.4% of the women in the misoprostol group and 2.9% in the sulprostone group, but 1.4% of the women in the misoprostol and 5.8% in the sulprostone group received medication for it. Hyperthermia, defined as a temperature of 38°C or more, occurred twice as frequently in the misoprostol group than in the sulprostone group (24.3% versus 11.6%; difference: +12.7%; 95% CI: +1.2% to +25.3%; *P* < .05). Hyperthermia occurred in 15.6% of women (7 of 45) who received less than 1000 *μ*g misoprostol, but in 40% (10 of 25) of those receiving 1000 *μ*g or more (*P* < .05).

We also considered the severity of side effects in a composite outcome consisting of either one of the following: hyperthermia, and women's report of either more than one episode of vomiting, severe nausea or moderate or severe diarrhea. This occurred in 30 women (42.9%) in the misoprostol group and 19 (27.5%) in the sulprostone group (difference: +15.3%; 95% CI: 0.0% to +31.0%; *P* > .05).

There was no difference between the misoprostol and sulprostone group in the use of analgesia and women's pain scores. There was a marked difference in women's reported lack of mobility (“some restriction” and “too restricted” combined) between the misoprostol (34.3%) and the sulprostone (63.8%) groups (difference: −29.5%; 95% CI: −13.6% to −45.4%; *P* < .05) in favor of misoprostol. Women's opinion on the acceptability of the procedure (82.9% in the misoprostol and 89.9% in the sulprostone group; difference: −7.0%; 95% CI: −18.3% to +4.4%), though, did not differ between the 2 groups. Overall satisfaction (91.4% in the misoprostol and 88.4% in the sulprostone group; difference: +3.0%; 95% CI: −7.0% to +13.0%), and preference for the other method (4.3% in the misoprostol and 8.7% in the sulprostone group; difference: −4.4%; 95% CI: −12.6% to +3.8%) were also slightly, but not significantly, in favor of misoprostol.

## 4. Discussion

When this study was initiated, sulprostone was well established in the Netherlands as a highly effective method for ending pregnancy after fetal death [14] but causing gastrointestinal side effects in 20%. We, therefore, required a substantial reduction in side effects to warrant the use of another prostaglandin analogue, misoprostol, which is not registered for obstetric use. As misoprostol is marketed for the prevention and treatment of gastroduodenal ulcers [17], it was anticipated that misoprostol would have less side effects with perhaps almost equal effectiveness. This did not materialize neither in the women's perception nor in the reports of their caregivers. If anything, all significant side effects combined were more frequent with misoprostol than with sulprostone. The only single significant difference related to hyperthermia and this occurred twice as frequently with misoprostol than with sulprostone. Notwithstanding this, during the trial clinicians clearly gained the impression that misoprostol was better tolerated and more effective than sulprostone. As a result there was increasing reluctance to randomize between the two treatments, which led to termination of the trial before its target was reached and before an objective assessment could be made. It would seem, therefore, that a number of intangible items colored the clinicians' perception. These may well include the ease of vaginal administration compared with the need for an intravenous line, women's perception of freedom of movement, and other factors that were not assessed. In comparison with the women themselves clinicians also tended to underestimate both the frequency and the severity of gastrointestinal side effects, but this occurred in both treatment groups and not to such an extent that it reached statistical significance.

In terms of effectiveness, we found no substantial differences between the two treatments as applied here (Table 2; Figure 2), although the median induction-delivery interval was 2 hours shorter with misoprostol than with sulprostone. In both groups a few women were switched to the alternative treatment, but this occurred less frequently than had been anticipated and more than 90% of women delivered within 36 hours, with no difference between treatments as allocated. While expulsion of the dead fetus occurred spontaneously in all women, there was a high incidence of placental retention and one third received either a manual removal or a curettage (Table 2). Incomplete expulsion, placental retention, and the need for curettage or manual exploration were equally common in both groups and clearly linked to the duration of pregnancy. Under 20 weeks of gestation, 60% of women had either placental retention for more than one hour or a uterine evacuation whilst this occurred in only 15% of those beyond 20 weeks.

A limitation of our study is that we cannot be certain to have used equipotent doses of both agents. While our sulprostone regimen was based on data from a previous controlled trial [14], no such information was available to determine the optimal dose of misoprostol. Even pharmacokinetic data on vaginal misoprostol were lacking at the time. Several such studies have become available since [18–22]. Although based on first trimester pregnancies and not entirely in agreement with each other, they are reasonably consistent in showing a large individual variation among women, with peak levels being reached on average about one hour after vaginal misoprostol administration, which then decrease gradually over a period of several hours. Low levels of misoprostol acid seem to remain present in serum for at least 6 hours, although uterine activity has mostly declined by that time [21]. Even now, dose regimens of misoprostol used for termination of pregnancy differ widely among published reports and there is little solid information that relates to intrauterine fetal death in particular [16, 23].

Yet, it has been known for more than 25 years [5] that prostaglandins and prostaglandin analogues have a higher uterotonic potency after fetal death than in live pregnancies of the same duration thereby permitting the use of lower doses [5]. This has been confirmed more specifically for misoprostol too [24, 25]. On the other hand, the occurrence of uterine hypertonus is less problematic in such pregnancies, which may explain why doses used to end pregnancy after fetal death have usually not been lower than those used for live pregnancies of similar duration. However, this approach may well increase the frequency of unpleasant side effects which form an additional burden for women who are heavily challenged already by the fetal demise. While we found no evidence for a difference in side effects between our different misoprostol regimens at different gestational ages, side effects and hyperthermia in particular did relate to the total doses used. It has been argued that the incidence of side effects correlates reasonably well with serum levels while the uterine effects seem to relate more to the duration than to the height of serum levels above a particular, but as yet not clearly defined, threshold [22].

There is only one other published comparison of misoprostol and sulprostone for terminating pregnancy after fetal death [25]. In that study vaginal misoprostol, 100 *μ*g every 12 hours, was compared with historical controls receiving intravenous sulprostone at 1 *μ*g per minute at gestational ages between 15 and 38 weeks. Whilst the median induction-delivery interval in the sulprostone group (12.3 hours) was identical to that observed in our study, in the misoprostol group it was 16.5 hours compared with 10.4 hours in our study. This, combined with more recent pharmacokinetic data [22], would suggest that the dose regimen used in that study was less adequate than ours, possibly because of the long interval between doses or the lack of differentiation between earlier and later gestations. Nevertheless, that study too concluded that misoprostol was the better of the two options with no significant differences in either effectiveness or side effects.

## 5. Conclusions

Our study found no evidence for a marked difference in side effects between the two treatments, although such difference seemed to exist in the clinicians' subjective perception during the course of the trial. There was no difference in the use of analgesia or in women's pain perception. Hyperthermia was more common in the misoprostol group. Both treatments scored the same in terms of gastrointestinal side effects, but, in the women's assessments, other elements of inconvenience associated with the induction of labor and especially freedom of movement were in favor of misoprostol. Although not formally assessed in this study, the cost differential is also largely in favor of misoprostol. Given that both treatments were equally effective, misoprostol would seem to be the preferred management option, albeit that further research may still establish more optimal dose regimens at different gestational ages than that used here.

## Figures and Tables

**Table 1:** Baseline characteristics (number) of the women included in the analysis.

Characteristic	Misoprostol	Sulprostone
	(*n* = 70)	(*n* = 69)
*Age (mean, SD)*	30.3 (5.2)	30.3 (4.8)
* * <20 years	2	1
* * ≥35 years	10	13

*Parity*		
* *Nulliparous	29	30
* *Parity ≥ 5	3	2

*Previous cesarean section*	4	4

*Previous miscarriages*		
* *None	48	46
* * ≥2	7	4

*Gestational age*		
* * <22 weeks	38	34
* *22–27 weeks	10	11
* *28–36 weeks	17	16
* * ≥37 weeks	4	6
* *Uncertain	1	2
* *Median	20.0	21.0
* *Range	14.5–40.0	14.0–40.5

*Duration of fetal death*		
* * <48 hours	21	19
* * ≥28 days	6	2
* *Inaccurate	28	31

Condition of cervix*		
* *Closed (dilatation 0 cm)	55/69	51/68
* *Length ≥ 3 cm	31/68	25/66
* *Length ≥ 2 cm	59/68	57/66

*Ruptured membranes*	5	1

*Fetal weight*		
* * <500 g	39	34
* *500–2,499 g	19	19
* * ≥2,500 g	8	6
* *Not recorded	4	10

*Cervical length or dilatation were not recorded for all women.

**Table 2:** Labor and delivery outcomes.

Outcome*	Misoprostol		Sulprostone	
	(*n* = 70)		(*n* = 69)	
	*n*	%	*n*	%
*Induction-delivery interval (hours)*				
* *Median	10.4		12.3	
* *Range	1.7–58.0		2.0 – 74.8	
* *10th–90th centile	4.5–22.0		5.8 – 31.3	

*Induction-delivery interval*				
* *Within 12 hours	41	58.6	34	49.3
* *Within 24 hours	64	91.4	59	85.5
* *Within 36 hours	68	97.1	64	92.8
* *Within 48 hours	69	98.6	67	97.1

*Switched to alternative treatment*	3	4.3	6	8.7

*Use of other uterotonics*	19	27.1	16	23.2
* *Before delivery	6		3	
* *After delivery	13		13	

*Placental retention*> 1* hour*	14	20.0	11	15.9

*Curettage or manual removal of placenta*	25	35.7	22	31.9

*Placenta delivered spontaneously within 1 hour without curettage or manual removal*				
* *At < 22 weeks	15/38	39.5	15/3	444.1
* *At 22–27 weeks	8/10	80.0	9/11	81.8
* *At ≥ 28 weeks	18/21	85.7	18/22	81.8
* *All women**	42/70	60.0	44/69	63.8

*Blood loss (mL)*				
* * <300 mL	43	61.4	43	62.3
* * ≥500 mL	15	21.4	14	20.3
* * ≥1,000 mL	5	7.1	6	8.6

*Blood transfusion*	1	1.4	3	4.3

*None of the differences between the two groups reaches statistical significance.

**Includes 3 women (1 misoprostol and 2 sulprostone) with too uncertain a duration of pregnancy to be classified in the gestational age categories.

**Table 3:** Frequency of side effects as recorded by the caregivers.

Outcome*	Misoprostol		Sulprostone	
	(*n* = 70)		(*n* = 69)	
	*n*	%	*n*	%
*Nausea and vomiting*				
* *None	53	75.7	54	78.3
* *All nausea/vomiting	5	7.1	8	11.6
* * * *Mild, unmedicated	2	2.9	2	2.9
* * * *Medicated	3	4.3	6	8.7
* *Not recorded	12	17.1	7	10.1

*Diarrhea*				
* *None	61	87.1	62	89.9
* *All diarrhea	7	10.0	7	10.1
* * * *Mild, unmedicated	6	8.6	3	4.3
* * * *Medicated	1	1.4	4	5.8
* *Not recorded	2	2.9	0	0

*Use of analgesia*				
* *None	12	17.1	10	14.5
* *Any pain relief	57	81.4	59	85.5
* * * *Mild analgesics	11	15.7	10	14.5
* * * *Opiates	14	20.0	12	17.4
* * * *Epidural	29	41.4	33	47.8
* * * *Opiates and epidural	3	4.3	4	5.8
* *Not recorded	1	1.4	0	0

*Hyperthermia* (≥38.0°C)*	17	24.3	8	11.6

*None of the differences are statistically significant except for the frequency of hyperthermia (Chi square test: *P* < .05).

**Table 4:** Women's assessment of the treatments and their side effects.

Outcome*	Misoprostol		Sulprostone	
	(*n* = 70)		(*n* = 69)	
	*n*	%	*n*	%
*Nausea*				
* *None	44	62.9	41	59.4
* *All nausea	23	32.9	25	39.1
* * * *Light,	14	20.0	17	24.6
* * * *Moderate	2	2.9	6	8.7
* * * *Severe	7	10.0	2	2.9
* *Not reported	3	4.3	3	4.3

*Vomiting*				
* *None	50	71.4	49	71.0
* *All vomiting	17	24.3	17	24.6
* * * *Only once	5	7.1	6	8.7
* * * *Several episodes	11	15.7	11	15.9
* *Not reported	4	5.7	3	4.3

*Diarrhea*				
* *None	56	80.0	56	81.2
* *All diarrhea	11	15.7	10	14.5
* * * *Light,	6	8.6	7	10.1
* * * *Moderate	4	5.7	1	1.4
* * * *Severe	1	1.4	2	2.9
* *Not reported	3	4.3	3	4.3

*Pain score (range 0 to 10)*				
* *Median (interquartile range)	5 (3–7)		5 (2–7)	

*Women's perception of the duration of induction*				
* *Too long	10	14.3	14	20.3
* *Not too long	26	37.1	26	37.7
* *Uncertain	31	44.3	26	37.7
* *Not answered	3	4.3	3	4.3

Women's opinion on restriction of movement*				
* *Felt no restriction	40	57.1	22	31.9
* *Any restriction	24	34.3	44	63.8
* * * *Some restriction	21	30.0	36	52.2
* * * *Too restricted	3	4.3	8	11.6
* *Not answered	6	8.6	3	4.3

*Women's opinion on the acceptability of the procedure*				
* *Very acceptable	34	48.6	23	33.3
* *Acceptable	24	34.3	39	56.5
* *Poor acceptability	7	10.0	4	5.8
* *Not answered	5	7.1	3	4.3

*Women's overall satisfaction*				
* *Very satisfied	50	71.4	50	72.5
* *Satisfied	14	20.0	11	15.9
* *Little or not at all	2	2.9	3	4.3
* *Not answered	4	5.7	5	7.2
*Would have preferred the other method*				
* *Yes	3	4.3	6	8.7
* *No	39	55.7	30	43.5
* *Do not know	23	32.9	30	43.5
* *Not answered	5	7.1	3	4.3

*There was no statistical difference in outcome between the two groups except for restriction of movement (Chi square test; *P* < .005).
